# Chemotherapy tips the scale in favour of cancer‐fighting B cells

**DOI:** 10.1002/ctm2.1407

**Published:** 2023-09-05

**Authors:** Ke Rui, Fan Xiao, Jie Tian, Jixiang Chen, Liwei Lu

**Affiliations:** ^1^ Department of Laboratory Medicine, Institute of Medical Immunology of Jiangsu University Affiliated Hospital of Jiangsu University Zhenjiang China; ^2^ Department of Pathology and Shenzhen Institute of Research and Innovation The University of Hong Kong Hong Kong China; ^3^ Centre for Oncology and Immunology Hong Kong Science Park Hong Kong China; ^4^ Department of Gastrointestinal Surgery Affiliated Hospital of Jiangsu University Zhenjiang China

1

The tumour‐specific immune response is crucial for the efficacy of conventional cytotoxic drugs. To date, the mechanisms underlying efficient T‐cell response triggered by cytotoxic drugs are not fully understood. In a newly published study in *Nature Medicine*, Lv et al. revealed how clinically efficient chemotherapy (i.e., gemcitabine, cisplatin) rewired the tumour tissue architectures in nasopharyngeal carcinoma (NPC).^1^ In this work, they identified a unique subset of innate‐like B cells (ILBs) that mainly accumulate in tertiary lymphoid organ‐like structures (TLSs), expand follicular helper (T_FH_) and helper type‐1 T (T_H_1) cells via the ICOSL–ICOS axis and eventually promote cytotoxic T lymphocyte (CTL) responses.^1^

The success of immune checkpoint therapy has revolutionised cancer management, as revealed by recent studies showing that various types of tumours can be efficiently treated by targeting immune components rather than solely by targeting malignant cells. There is increasing evidence that conventional treatment modalities, in addition to direct tumour killing, can also exert immunomodulatory effects.^2^ In this new work,^1^ Lv et al. found that efficient chemotherapy substantially reshaped composition and functionality of tumour immune infiltrates towards antitumour microenvironment in NPC, where antigen‐presentation capacity of tumour cells was enhanced, accompanied by increased effector T cells, while immunosuppressive elements were substantially eliminated. Intriguingly, the rewired antitumour network did not solely consist of pre‐existing tumour‐infiltrating T lymphocytes; it contained large amounts of novel clonotypes that had not been detected in pre‐treatment counterparts. These interesting findings suggest that efficient chemotherapy exerts not only cytotoxic effects on tumour cells but also immunomodulatory effects on tumour immune microenvironment (TIME), which seems to ‘hit’ the existing suppressive TIME and reestablish the ‘brand‐new’ effector T‐cell network. However, in progressive tumours unresponsive to chemotherapy, profoundly increased immunosuppressive SPP1^+^ macrophages and exhaustive T cells were identified in metastatic liver from colorectal cancer patients.^3^ These reports highlight that TIME exhibits marked therapy‐induced plasticity and that a distinct cellular ecosystem evolved after chemotherapy elicits an antitumour response.

T lymphocytes play a central role in antitumour immunity that leads to the eradication of tumour cells. Dendritic cells (DCs) can engulf, process and present tumour antigens to prime T cells, which have been recognized as a major player in promoting T‐cell responses. Remarkably, Lv et al. uncovered that the post‐chemotherapy effector T‐cell response was not driven by conventional DCs, but by a subset of B cells that served as the core determinant. In addition to other conventional antigen‐presenting cells (APCs), B cells have been found to act as APCs for decades. In the models of influenza infection, B cells have been shown to be essential and sufficient for the activation of T cells.^4^ However, whether B cells could serve as APCs to drive T‐cell responses in TIME remains largely unclear. Consistent with the findings by Lv et al., two other studies have reported that B cells can elicit and support antitumour T‐cell responses.^5^, ^6^ Cui et al. found that neoantigen‐specific B cells initiated T_FH_ to promote CTL effector functions in a mouse model of lung cancer.^5^ Lu et al. showed that complement signaling‐induced B cells boosted antitumour immunity by enhancing effector T cell to regulatory T cell ratio in breast cancer.^6^ These findings support a role of B cells as APCs in TIME. Compared with other conventional APCs, the role of B cells as APCs in tumours has been much less appreciated, which is probably attributed to their relative insufficiency in taking up antigens, or rare infiltration when compared with their antigen‐specific counterparts in steady states. However, as shown in the work by Lv et al., B cells far outnumber DCs in TIME, enabling them to interact with T cells with a geographical advantage in situ. Moreover, their binding affinity can be dramatically enhanced upon antigen encounter, making them more effective in antigen binding at lower concentrations than DCs. Thus, further studies are needed to elucidate the antigen‐presenting role of B cells and modulate such function to enhance antitumour T cells for effective tumour treatment.

B cells are highly heterogeneous population. It has been reported that interleukin (IL)‐10‐producing CD24^+^CD38^hi^ regulatory B (Breg) cells significantly restrain T_FH_ responses in the model of primary Sjögren's syndrome.^7^ Similarly, in tumour microenvironment, IL‐10‐producing Breg with a unique PD1^hi^CD5^hi^CD24^−/+^CD27^hi/+^CD3^dim^ phenotype also elicited immunosuppressive functions and led to tumour progression in hepatocellular carcinoma.^8^ In addition to serving as an immune regulator, B cells are also important executors of humoural immunity for their ability to produce diverse high‐affinity antibodies upon differentiating into plasma cells. Plasma cells have been considered to play a protective role during tumour initiation. However, in TIME, IgG^+^ plasma cells are found to create immunosuppressive environment by activating protumourigenic macrophages in hepatocellular carcinoma.^9^ Interestingly, B cells mainly exhibit an immature phenotype in NPC and high expression of B‐cell signature genes is associated with favourable prognosis, as shown in previous work by the same team.^10^ In the new work, this team goes one step further and elucidates that chemotherapy can induce a novel subset of cancer‐fighting ILB from naïve B cells. Different from conventional follicular B cells that require help from T cells and mature into plasma cells, ILBs appear to adopt different developmental pathways, as they are activated upon innate stimulation (i.e., through pattern recognition receptors of Toll‐like receptor family) and maintain natural IgM levels at steady state. It is also interesting to note that ILBs are not randomly distributed in post‐chemotherapy TIME, but form interactive niches with T lymphocytes in TLSs that may further enhance their interactive cross‐talks. Together, these findings support the notion that not only the cytokine production or antibody secretion nature of B cells per se but also their heterogeneous differentiation status is one of the determinants of multifaceted functions of B cells.

In summary, the newly published work by Lv et al. has significantly advanced our current understanding of the immunomodulatory effect of chemotherapy, which also opens a new avenue to bridge among B cells, innate sensing, and TLSs in affecting antitumour immunity and brings B cells to the forefront for sustaining the immunomodulatory effect of chemotherapy.

## CONFLICT OF INTEREST STATEMENT

The authors declare they have no conflicts of interest.
